# Interventions for common mental disorders in the occupational health service: a systematic review with a narrative synthesis

**DOI:** 10.1007/s00420-020-01535-4

**Published:** 2020-04-03

**Authors:** Iben Axén, Elisabeth Björk Brämberg, Marjan Vaez, Andreas Lundin, Gunnar Bergström

**Affiliations:** 1 Unit of Intervention and Implementation Research for Worker Health, Institute of Environmental Medicine, Karolinska Institutet, 171 77 Stockholm, Sweden; 2 Department of Public Health and Community Medicine, Sahlgrenska Academy, University of Gothenburg, Box 414, 405 30 Gothenburg, Sweden; 3 Division of Insurance Medicine, Department of Clinical Neuroscience, Karolinska Institutet, 171 77 Stockholm, Sweden; 4 Department for Public Health Sciences, Karolinska Institutet, 171 77 Stockholm, Sweden; 5 Department of Occupational Health Sciences and Psychology, University of Gävle Centre for Musculoskeletal Research, Kungsbäcksvägen 47, 801 76 Gävle, Sweden

**Keywords:** Common mental disorders, Occupational health service, Workability

## Abstract

**Introduction:**

Common mental disorders (CMD) are leading causes of decreased workability in Sweden and worldwide. Effective interventions to prevent or treat such disorders are important for public health.

**Objective:**

To synthesize the research literature regarding occupational health service (OHS) interventions targeting prevention or reduction of CMD among employees. The effect on workability (sickness absence, return-to-work and self-reported workability) and on CMD symptoms was evaluated in a narrative analysis.

**Data sources:**

The literature search was performed in four electronic databases in two searches, in 2014 and in 2017.

**Eligibility criteria (using PICO):**

Population: studies investigating employees at risk or diagnosed with CMD, as well as preventive workplace intervention targeting mental health. Intervention: studies where the recruitment or the intervention was delivered by the OHS or OHS personnel were included. Control: individuals or groups who did not receive the target intervention. Outcome: all types of outcomes concerning sickness absence and psychological health were included.

Study quality was assessed using a Swedish AMSTAR-based checklist, and results from studies with low or medium risk of bias were narratively synthesized based on effect or absence thereof.

**Results:**

Thirty-three studies were included and assessed for risk of bias. Twenty-one studies had low or medium risk of bias. In 18 studies, rehabilitation interventions were evaluated, 11 studies concerned interventions targeting employees at risk for developing CMD and four studies investigated preventive interventions. Work-focused cognitive behavioral therapy and problem-solving skill interventions decreased time to first return-to-work among employees on sick leave for CMD in comparison with treatment-as-usual. However, effect on return to full-time work was not consistent, and these interventions did not consistently improve CMD symptoms. Selective interventions targeting employees at risk of CMD and preventive interventions for employees were heterogeneous, so replication of these studies is necessary to evaluate effect.

**Limitations:**

Other workplace interventions outside the OHS may have been missed by our search. There was considerable heterogeneity in the included studies, and most studies were investigating measures targeting the individual worker. Interventions at the workplace/organizational level were less common.

**Conclusions and implication of key findings:**

Return-to-work and improvement of CMD symptoms are poorly correlated and should be addressed simultaneously in future interventions. Further, interventions for CMD administered through the occupational health service require further study. Rehabilitative and preventive strategies should be evaluated with scientifically robust methods, to examine the effectiveness of such interventions.

## Background

Common mental disorder is a term incorporating depression, anxiety, adjustment disorders, and stress-related ill health, all of which have major consequences around the world. For the individual, CMD causes suffering, pose a risk of social isolation, and threaten the personal income. In medium- to high-income countries, depression has become the diagnosis with the highest societal burden due to disability, decreased workability, and years lost to premature death (https://osha.europa.eu/en/themes/psychosocial-risks-and-stress). Individuals on long-term sick leave due to CMD are also at a higher risk for other types of mortality such as cardiovascular disease and cancer, and depressed individuals have a higher risk for suicide (Bryngelson et al. 2013).

In Sweden, CMD have become the leading causes for sickness absence with benefits. In 2016, 38% of all new sickness benefit episodes were due to CMD (Social Insurance Agency of Sweden 2016). Further, sickness absence periods due to CMD are longer and recurrent compared to other diagnosis, and are also more prevalent among women (Swedish Social Insurance Agency 2014). In a report from the Organisation for Economic Co-operation and Development (OECD) from 2018, costs for CMD, summarizing costs for health care, sickness absence insurance and lost productivity, were estimated at 21 billion euro in Sweden in 2015 (OECD and EU 2018).

The risk of CMD is lower among individuals who are working compared to those who are not gainfully employed (Waddell 2006). However, some risk factors for CMD are work related, although different between professions and socioeconomic groups (Marmot 2017). Such risk factors include high demands, low control, poor social support, and an effort-reward imbalance (Theorell et al. 2015; Aronsson et al. 2017). Protective occupational factors, on the other hand, include experiencing fair treatment and having influence over one’s work. Moreover, 40% of employees in European workplaces feel that work-related stress is not handled well (https://osha.europa.eu/en/themes/psychosocial-risks-and-stress).

To date, many rehabilitative interventions for CMD, like cognitive behavioural therapy (CBT), physical activity, and relaxation have focused on symptoms (Nieuwenhuijsen et al. 2014). Further, such interventions are directed towards the worker, placing the responsibility of improving the health status on the individual. Less commonly, organizational change (Joyce et al. 2016) is used as interventions to improve workers’ mental health. Moreover, improvement in symptoms does not necessarily translate into return-to-work (RTW) (Ejeby et al. 2014). Adding a workplace-directed intervention to a clinical intervention seems to decrease sick leave and facilitate RTW (Nieuwenhuijsen et al. 2014). In a review from 2012, interventions successful also in RTW were work directed and based on problem-solving therapy (PST) (Arends et al. 2012). PST interventions include identifying the problem and possible solutions applied in the work environment, as well as developing strategies to address the identified problems.

The occupational health service (OHS) exists in most industrialized countries (Rantanen et al. 2017) and is, by definition, working to promote employee health. The OHS may act in the preventive field to ensure that ill health is prevented or minimized, as well as having a role in facilitating RTW through rehabilitation and work adaptations when ill health has occurred (Joosen et al. 2015). As the OHS is operating in the workplace setting, knowledge about the specific work situation is good, and investigations and interventions can be directed appropriately both on an individual, group, and organizational level. Due to the limited knowledge concerning effects on workability (for instance sickness absence, sickness presence, and RTW) from interventions given to employees with CMD, and that many interventions appear not to be work oriented, it would be important to synthesize intervention research where the OHS is involved (Verbeek 2007).

To the best of the authors’ knowledge, no systematic mapping of OHS interventions concerning CMD exists. Consequently, the aim of this systematic review was to synthesize the scientific research on OHS interventions targeting prevention or reduction of CMD at the workplace, using the outcomes of workability and symptoms of CMD.

## Method

### Eligibility criteria

Eligibility was assessed according to the studies’ population, intervention, control, and outcome (PICO system) as follows:

#### Population

Studies investigating employees (individuals, groups, or organizations) at risk for CMD (as identified by a risk tool) or diagnosed with CMD were included. Studies on employees at workplaces evaluating stress preventive interventions were also included. Studies investigating individuals with severe mental disorders (like schizophrenia) were excluded.

#### Intervention

Studies where the recruitment or the intervention was delivered by the OHS or OHS personnel were included. Despite diversity, we included all types of OHS if they were labelled as such. Any type of intervention to prevent or reduce the risk of CMD or consequences thereof on an individual or at the organizational level was included. Longitudinal studies with baseline and follow-up measurements were included (as described in the PICOs). Studies where it was not possible to clearly understand the intervention through reading were excluded.

#### Control

Individuals or groups who did not receive the target intervention.

#### Outcome

All types of outcomes concerning sickness absence, including RTW, and psychological health were included. In the following, we use the term “workability” as a summarizing term including sickness absence, RTW, and self-reported workability. This included workers who had the ability to work part time.

Eligible study designs were randomized controlled trials, quasi-experimental studies, and longitudinal studies with baseline and follow-up measurements. Systematic reviews were also included. Publications written in English, Danish, Norwegian, or Swedish were accepted.

### Search strategy and information sources

A systematic search strategy was developed in collaboration with librarians and adapted to the following four electronic databases; Medline, PsycINFO, Web of Science, and Cochrane CENTRAL. The original search period was January 1990 to March 2014. A second search for papers published between April 2014 and May 2017 was added using the same search terms. Search terms were developed and refined in discussions between GB and the librarian. The final terms were “Occupational Health Services”, “Occupational Disease”, “Occupational Health”, “Occupational Medicine”, “Occupational Health Nursing”, “Occupational Health Physicians”, “Occupational Injuries”, “Return to Work”, “Workplace”, “Clinical Trial”, “Comparative Study”, “Evaluation Studies”, “Meta Analysis”, “Multicenter Study”, “Observational Study”, “Review”, “Systematic Reviews”, “Epidemiologic Studies”, “Intervention Studies”, “Mental Disorders”, “Stress”, “Burnout”, “Depression”, “Anxiety”, “Adjustment Disorders” and combinations thereof. Abstracts were downloaded onto an Endnote × 7 library. In a second step, the reference lists of the included studies were searched for additional studies, and articles included in reviews were also checked for eligibility.

### Selection assessment and data extraction

Titles and abstracts of retrieved records were evaluated by two pairs of researchers, MV and AL for the first search in 2014, IA and EBB for the second search in 2017. In each round, the pair met and reviewed 100 abstracts together to calibrate their judgement, after which the remaining abstracts were divided between the reviewers. They each decided on inclusion and exclusion. If in doubt, the abstract was included.

Review of the full texts started in the same way by the pair meeting and reviewing 30 full texts together. In cases of disagreement, the articles were discussed until agreement was reached. Again, the list of articles was divided between the reviewers to individually decide on which to include and exclude. When in doubt, the study was discussed between the pair until agreement was reached.

### Quality assessment

All reviewers independently completed a quality assessment of the included studies, using a review protocol developed by the Swedish Agency for Health Technology Assessment and Assessment of Social Services (SBU), (https://www.sbu.se/contentassets/997d90960dbf40bbb4a1121d32c8acda/bilaga_3_granskningsmallar.pdf) for systematic reviews based on AMSTAR (Shea et al. 2007). This checklist is in Swedish, which made communication between reviewers easy. The quality assessment focused on methodological limitations that may introduce bias and run the risk of jeopardizing the study results. In a second meeting, the pair’s assessments were compared and disagreements solved through discussion. The included studies were rated as low, medium, or high risk of bias. Disagreements were resolved by consensus with GB during the entire quality assessment process.

### Data synthesis

Data concerning year of publication, country of origin, type of intervention and control, outcome measure, time of follow-up, and result (positive or negative) were summarized. All studies were categorized according to their outcomes; first according to their effect on workability and second according to their effect on CMD symptoms. Lastly, based on the quality assessment, the results from studies deemed as having low and medium risk of bias were collated in a narrative summary to assess if studies examining the same intervention reported similar effect on one of the outcome categories. Studies with a high risk of bias were not considered in the summary as results from such a study may be distorted (reflect effects from factors other than the intervention). If a minimum of two studies of medium or low risk of bias were evaluating the intervention, the scientific quality was deemed satisfactorily, and conclusions could be drawn. However, no heterogeneity between the included studies was a prerequisite for this judgement. If only single studies with medium risk of bias evaluated an intervention, no conclusions were made as the scientific quality was deemed uncertain.

The results across studies were simply synthesized according to (i) effect or (ii) no evidence of effect.

## Results

**Fig. 1:**
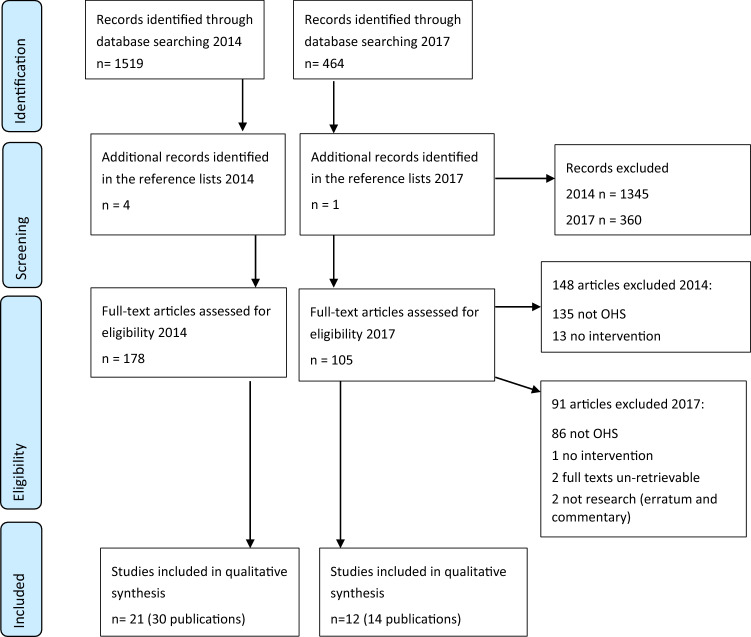
The searches performed in 2014 and 2017, exclusions and final inclusions

The initial searches resulted in 1983 abstracts, and based on the first screening of abstracts, 1705 were excluded. Reviews of the remaining 278 full texts and an additional 5 found by searching the reference lists, led to the exclusion of another 239 articles, leaving 44 scientific articles, representing 33 original studies. The reason that the number of articles/publications was higher than the number of studies is that some studies were reported in more than one article. The search is summarized in Fig. 1.

### Characteristics of the included studies

Seventeen studies were from the Netherlands, four were from Finland, three were from Sweden, three were from Japan, two were from the United Kingdom, and one study was conducted in each of the following countries: Denmark, Canada, Germany, and Korea.

In total, the studies included 11,504 study subjects, ranging between 24 and 3379 individuals. The quality assessment ranked 6 studies as low risk of bias, 16 as medium, and 11 as high risk of bias.

The majority of the studies (*n* = 18) reported rehabilitative interventions targeting individuals on sick leave due to CMD, 11 studies reported interventions directed towards individuals at risk of developing CMD, i.e. the intervention was based on a screening tool to identify workers at risk and directed towards this specific group, and a four studies were classified as preventive interventions directed towards individuals and organizations, without regarding individual risk status. Twenty-five of the studies were randomized control trials (RCTs), six were observational studies, one was a mixed method study (including an observational component) and one was a systematic review. Finally, the studies reported different outcome measures, usually measures of workability, mainly sickness absence and RTW, and symptoms (e.g. depression, anxiety, stress, insomnia). A summary description of the studies is presented in Appendix 1.

#### Rehabilitative interventions of individuals with CMD

A total of 18 studies reported rehabilitation interventions directed towards employees with CMD. In some of these studies, the employees were on sick leave for a CMD diagnosis. Six of the studies were classified as low risk of bias, eight as medium risk of bias, and four were assessed as having high risk of bias.

Cognitive behavioral (CBT) and problem-solving therapy (PST) interventions were used in six of these studies and in one meta-analysis. PST is based on CBT, and, therefore, we decided a priori to group these studies together. All of these studies included employees who were on sick leave due to CMD and also reported an outcome related to workability and are summarized in Table 1.

**Table 1:** Studies investigating cognitive behavioral (CBT) and problem-solving therapies (PST) provided by the occupational health services (OHS) as rehabilitative interventions for individuals on sick leave due to CMD

Author, year of publication, type of study, country and number of subjects included (reference in the text)	I = intervention C = control	Follow up time Result on workability	Follow up time Result on symptoms	Risk of bias
Volker et al. (2015) RCT The Netherlands *N* = 220	I: E-health modules based on CBT, PST-based interventions, and decision support to OHS physician C: treatment as usual	12 months: time to first day RTW + Time to full RTW 0	12 months: severity of depression 0	Low
Arends et al. (2013) RCT The Netherlands *N* = 158	I: PST with prevention of recurrence C: treatment as usual	12 months: repeated sickness absence + Cost effectiveness 0 Time to repeated sickness absence +	12 months: psychological ill-health 0	Low
Rebergen et al. (2009) RCT The Netherlands *N* = 240	I: PST C: minimal involvement of OHS physician, referral to psychologist	12 months: time to (partial or full) RTW 0 Costs for care + Production loss reduction 0	NR^a^	Low
Van der Klink et al. (2003) RCT The Netherlands *N* = 192	I: PST C: treatment as usual	12 months: time to partial RTW + Duration of sick-leave + Time to full RTW 0	3 and 12 months: depression 0	Low
Doki et al. (2015) meta-analysis Japan All included publications from the Netherlands, except one from Denmark *N* = 1554	I: PST intervention or CBT given at the OHS C: treatment as usual	4–18 months: number of days with sickness absence all employees + Subgroup 1^b^: number of days with sickness absence all employees 0 Subgroup 2^b^: number of days with sickness absence all employees 0	NR^a^	Medium
Vlasveld et al. (2012,2013) RCT The Netherlands *N* = 126	I: PST, self-help, work- place intervention C: treatment as usual	12 months: time to full time work 0	12 months: Time to ≥ 50% decrease in depression + other measures of depression 0	Medium
Kröger et al. (2015) Controlled, matched study Germany *N* = 26	I: CBT with focus on RTW C: CBT	12 months: sickness absence +	12 months: depression 0	High

A positive effect is shown by + , no effect by 0

^a^Outcome not reported

^b^Subgroup 1 = employees on sick leave due to CMD, subgroup 2 = employees not on sick leave due to CMD

Based on studies with low or medium risk of bias, PST/CBT interventions were shown to be effective in decreasing days with sickness absence (Doki et al. 2015) as well as duration of sick leave (Klink et al. 2003) and recurrent sick leave (Arends et al. 2013). One study demonstrated a reduction of days to RTW (Volker et al. 2015), but these interventions were generally not found to improve full time RTW (Volker et al. 2015; Rebergen et al. 2009; Van der Klink et al. 2003; Vlasveld et al. 2012, 2013) after 12 months follow-up among individuals with CMD.

PST/CBT interventions did not significantly improve symptoms of CMD (Arends et al. 2013; Klink et al. 2003; Vlasveld et al. 2012, 2013) in most instances, only in one study (Vlasveld et al. 2012, 2013) where depression symptoms were reduced with > 50%).

Other studies examined interventions from the OHS physician (who were trained in guideline use or could consult a psychiatrist), a participatory intervention, multi-professional rehabilitation, a self-help manual coupled with PST, a workplace and pharmacological intervention, stress management, a gradual exposure to stress and occupational therapy. These studies are summarized in Table 2. Eight studies were assessed as low or medium risk of bias.

**Table 2:** Studies investigating different interventions [other than cognitive behavioral (CBT) and problem-solving therapies (PST)] for employees with CMD

Author, year of publication, type of study, country, and number of subjects included (reference in the text)	I = intervention C = control	Follow-up time Result on workability	Follow-up time Result on symptoms	Risk of bias
Van Beurden et al. (2017) RCT The Netherlands *N* = 3379	I: intervention from OHS physician who was trained in treatment guidelines C: treatment as usual	12 months: time to full time RTW 0 Time to first day of RTW 0 Sickness absence(hours) 0		Low
Van Beurden et al. (2015) RCT The Netherlands *N* = 128	I: intervention from OHS physician who was trained in treatment guidelines C: treatment as usual		3 months: workers self-efficacy beliefs to RTW +	
Van Oostrom et al. (2009, 2010) RCT The Netherlands *N* = 145	I: participatory intervention + treatment as usual C: usual treatment in guidelines	12 months: time to full RTW 0	12 months: depression 0 Anxiety 0 Stress 0	Low
Valtonen et al. (2015) RCT Finland N = 283	I: multi professional rehabilitation program C: treatment as usual	NR^a^	12 months: sense of coherence (SOC) 0	Medium
Goorden et al. (2014) RCT The Netherlands *N* = 126	I: self-help manual, problem-based intervention, workplace intervention, and pharmacological intervention as needed C: treatment as usual	12 months: cost effectiveness 0	12 months: quality of life 0	Medium
Dalgaard et al. (2014) RCT Denmark *N* = 137	I: stress handling and a workplace intervention C: no treatment was offered in the study; participants were free to seek care	NR^a^	10 months: sleep problems 0 Decreased cognitive ability 0	Medium
Noordik et al. (2013) RCT The Netherlands *N* = 160	I: gradual increased exposure to stress at work C: usual treatment in guidelines	12 months: RTW –	12 months: depression 0 Anxiety 0 Stress 0	Medium
Hees et al. (2013) RCT The Netherlands *N* = 117	I: occupational therapy C: treatment as usual	18 months: RTW 0	18 months: depression +	Medium
Van der Feltz-Cornelis et al. (2010) RCT The Netherlands *N* = 60	I: consultation with OHS physician who consults a psychiatrist C: consultation with OHS physician without contact with a psychiatrist	3 months: RTW (full Time) + 6 months time to RTW 0	6 months: depression 0 Anxiety 0	Medium
Bender et al. (2016) mixed methods Canada *N* = 141	I: educational intervention to increase awareness of symptoms related to stress or trauma, multi-disciplinary treatment program and coordinated RTW C: treatment as usual	6 months: RTW –	6 months: symptoms related to posttraumatic stress-syndrome 0	High
Grossi and Santell (2009) Observational study Sweden *N* = 24	I: stress management 3 months C: treatment as usual	6 And 12 months: sickness absence 0	6 And 12 months: depression 0 Exhaustion 0	High
de Vente et al. (2008) RCT The Netherlands *N* = 82	I1: individual stress management I2: group-based stress management C: treatment as usual	10 months: sickness absence 0	10 months: decrease in symptoms (depression, anxiety, exhaustion) 0	High

A positive effect is shown by + , no effect by 0, negative effects by –

^a^Outcome not reported

Generally, these interventions did not significantly improve full time RTW (Van Beurden et al.  2015, 2017; Oostrom et al. 2009; Noordik et al. 2013; Hees et al. 2013; Feltz-Cornelis et al. 2010) after 3–18 months follow-up among individuals with CMD. The only exception was that educating the physician to work with the psychiatrist (Feltz-Cornelis et al. 2010) which showed a statistically significant effect on RTW after 3 but not after 6 months.

Depression symptoms were found to be significantly improved by occupational therapy (Hees et al. 2013) but not by any other investigated intervention (Oostrom et al. 2009, 2010; Noordik et al. 2013; Feltz-Cornelis et al. 2010). One study found increased self-efficacy beliefs in RTW after the OHS physician followed guideline recommendations (Beurden et al. 2015). Symptoms of stress were not improved by any of the investigated interventions (Oostrom et al. 2009; Noordik et al. 2013), and neither was anxiety (Oostrom et al. 2009; Noordik et al. 2013; Feltz-Cornelis et al. 2010), sense of coherence (Valtonen et al. 2015) or sleep problems and cognitive ability (Dalgaard et al. 2014).

#### Interventions directed towards employees at risk for CMD

In eleven studies, interventions were directed towards individuals labeled as “at risk” of future CMD based on results from screening questionnaires. Six of these were assessed as medium and 5 as high risk of bias.

Again, CBT was used in some of the interventions. These studies are summarized in Table 3.

**Table 3:** Studies investigating CBT directed towards individuals with a risk of CMD

Author, year of publication, type of study, country, and number of subjects included (reference in the text)	I = intervention C = control	Follow-up time Result on workability	Follow-up time Result on symptoms	Risk of bias
Yamamoto et al. (2016) Japan RCT *N* = 130	I: group sessions for CBT treatment of insomnia C: waiting list	NR^a^	3 months: emotional stress 0 Insomnia 0	Medium
Lexis et al. (2011) RCT The Netherlands *N* = 139	I: CBT, 10–12 sessions C: treatment as specified in guidelines	12 months: sickness absence + 18 months: sickness absence 0	6–12 months: depression +	Medium
Grime et al. (2004) RCT United Kingdom *N* = 48	I: E-CBT + treatment as usual C: treatment as usual	NR^a^	0–1 months: depression and anxiety + 3–6 Months: depression and anxiety 0	High

A positive effect is shown by + , no effect by 0

^a^Outcome not reported

Regarding workability; in studies of low or medium risk of bias where depressive symptoms improved, sickness absence improved after 12 months, but not after 18 months Valtonen et al. (2015).

Conclusions based on studies with low or medium risk of bias showed one study with positive effects on depression (Lexis et al. 2011), but the other failed to show statistically significant effects on emotional stress and insomnia (Yamamoto et al. 2016).

The remaining eight studies examined interventions including consulting a physician, advice, and personal feedback. These studies are presented in Table 4. Six studies were assessed as having medium risk of bias, and three as having a high risk of bias.

**Table 4:** Studies investigating physician consultation, stress handling with mindfulness, advice, and personal feedback directed towards individuals with a risk of CMD and psychoanalytical therapy

Author, year of publication, type of study, country, and number of subjects included (reference in the text)	I = intervention C = control	Follow-up time Result on workability	Follow-up time Result on symptoms	Risk of bias
Noben et al. (2015) RCT The Netherlands *N* = 413	I: screening for psychological ill health, personal feedback, visit with OHS physician C: treatment as usual	6 months: decreased costs due to increased productivity + Net value (Sickness absence and presentism) +	NR^a^	Medium
Noben et al. (2014) RCT The Netherlands *N* = 617	I 1: screening for psychological ill health, personal feedback, visit with OHS physician I 2: screening for psychological ill health personal feedback, e-intervention C: treatment as usual	6 months: cost related to the intervention I 1 + Cost related to the intervention I 2 +	NR^a^	Medium
Kilfedder et al. (2010) Karatzias et al. (2011) RCT United Kingdom *N* = 90	I 1: face-to-face consultation about stress and help to identify stressors I 2: telephone consultation with same content C: bibliotherapy (book info and task)	NR^a^	4 months: core psychological health 0 Ghq-12 psychological ill health + Stress 0	Medium
Peterson et al. (2008) RCT Sweden *N* = 131	I: collegial talks C: nothing	NR^a^	12 months: exhaustion 0 Anxiety 0 Depression 0 General health +	Medium
Kant et al. (2008) RCT The Netherlands *N* = 299	I: preventive talks with OHS physician C: treatment as usual	12 months: total sick leave 0 Long-term sickness absence 0 *Modified analysis* Total sick leave + Long-term sickness absence 0	NRa	Medium
De Boer et al. (2004) RCT The Netherlands *N* = 116	I: consultations with OHS physician and a work plan C: treatment as specified in guidelines	24 months: early retirement + Sickness pension 0 Workability 0	24 months: exhaustion 0 Quality of life 0	Medium
Kuoppala and Kekoni (2013) observational study Finland *N* = 52	I: stress handling with mindfulness, meditation	NR^a^	6 Months: depression + Anxiety 0 Burnout 0 Exhaustion +	High
Gärtner et al. (2013) Ketelaar et al. (2013) RCT The Netherlands *N* = 379	I: preventive talks with OHS physician C: treatment as usual	6 months: health care utilization + Workability +	6 months: depression 0 Anxiety 0	High
Salmela-Aro et al. (2004) RCT Finland *N* = 98	I 1: psychoanalytical group therapy I 2: psychodrama C: talks with physician/psychtherapist	NR^a^	12 months: exhaustion + Work-related personal activities +	High

A positive effect is shown by + , no effect by 0, negative effect by −

^a^Outcome not reported

Sickness absence and presentism were reduced in one study (Noben et al. 2015), the risk of early retirement was reduced in another (Boer et al. 2004) and a reduction of total sickness absence was found in one study (Kant et al. 2008).

Five of the studies had medium risk of bias and investigated the effect on CMD symptoms but failed to show any improvements in these outcomes. However, some health benefits were seen in two studies (Karatzias et al. 2011; Peterson et al. 2008).

#### Preventive interventions

Four studies investigated preventive interventions directed towards individuals and organizations to prevent CMD. One was assessed as having medium risk of bias and three as having high risk of bias. These studies are summarized in Table 5

**Table 5:** Studies investigating interventions aimed at preventing CMD

Author, year of publication, type of study, country, and number of subjects included (reference in the text)	I = intervention C = control	Follow-up time Result workability	Follow-up time Result on symptoms	Risk of bias
Vuori et al. (2012) Ahola et al. (2012) RCT Finland *N* = 718	I: course in career development C: written information on career opportunities	7 months: wanting to retire +^b^	7 months: depression +^b^ Exhaustion 0 Mental resources +^b^	Medium
Vinberg et al. (2015) Longitudinal study with panel data Sweden *N* = 311	I: Individual- and group-based interventions, ex physical activity, health measurements, supervision C: no intervention	NR^a^	1 And 2 years: health and psychosocial work environment 0 Tiredness at work + Worry over one’s ability to handle work tasks +	High
Kim et al. (2014) 2014 Observational study Korea *N* = 211	I: stress-handling program C: no intervention	NR^a^	2 months: workers: Work-related stress + Symptoms of stress 0 Administrators: Work-related stress 0 Symptoms of stress 0	High
Kobayashi et al. (2008) Observational study Japan *N* = 1071	I: workshop on an action plan to improve the work environment, participation C: no intervention	12 months: sickness absence 0	12 months: vitality +^c^ Depression +^c^ Irritation 0 Tiredness 0 Anxiety 0	High

A positive effect is shown by + , no effect by 0

^a^Outcome not reported

^b^No effect when controlling for depression at study start

^c^Effect among women, but not among men

In the study with a medium risk of bias (Vuori et al. 2012), a course in career development designed to prevent CMD, showed no effects on either wanting to retire, on symptoms of CMD, or on mental resources after controlling for depression at study start.

## Discussion

A systematic review with a narrative synthesis was conducted in the specific setting of occupational health service, as this is a potentially important context for work-directed interventions to prevent or reduce symptoms or to improve workability among employees with CMD. Interventions based on PST and work-focused CBT were the most frequently studied interventions, used in rehabilitation and interventions directed at employees at risk.

The studies were rather heterogeneous concerning which interventions were used and which outcomes were measured, as well as the intent of the intervention; prevention, at risk or rehabilitation. The majority of the studies concerned rehabilitation of individuals who were diagnosed with CMD (Volker et al. 2015; Arends et al. 2013; Rebergen et al. 2009; Klink et al. 2003; Doki et al. 2015; Vlasveld et al. 2012,2013; Kroger et al. 2015; Beurden et al. 2015,2017; Oostrom et al. 2009,2010; Valtonen et al. 2015; Goorden et al. 2014; Dalgaard et al. 2014; Noordik et al. 2013; Hees et al. 2013; Feltz-Cornelis et al. 2010; Bender et al. 2016; Grossi and Santell 2009; Vente et al. 2008). In terms of workability, there were some evidence of improvement in time to first return-to-work among employees on sick leave for CMD receiving work-oriented PST and CBT, in comparison with treatment-as-usual. However, these interventions failed to improve full time RTW. Generally, symptoms decreased both among employees receiving interventions or treatment as usual, but usually there were no statistically significant differences between interventions and control in this respect.

An alternative or complementary strategy to rehabilitation can be to address the problem before it gets to the stage where large numbers of individuals suffer from CMD, i.e. to implement effective interventions directed towards people at risk of developing such problems. Using validated screening tools to identify these individuals, early interventions could be put in place. Eleven studies were directed towards employees at risk of developing CMD (Lexis et al. 2011; Grime 2004; Noben et al. 2014,2015; Kilfedder et al. 2010; Karatzias et al. 2011; Peterson et al. 2008; Kant et al. 2008; Boer et al. 2004; Kuoppala and Kekoni 2013; Gartner et al. 2013; Ketelaar et al. 2013; Salmela-Aro et al. 2004). The interventions included a great variety of measures, but the evidence is not sufficient to recommend a specific type or timing of interventions. We conclude that more research is needed.

Studies investigating preventive interventions were few (Vuori et al. 2012; Ahola et al. 2012; Vinberg et al. 2015; Kim et al. 2014; Kobayashi et al. 2008) and generally carried a high risk of bias. It would have been interesting to examine the effect of systematic work-environment measures aiming to create workplaces where people would get support, have control, and adequate time for recuperation. As only one study with sufficient quality was included in our review (Ahola et al. 2012), conclusions regarding effect of preventive interventions remain uncertain.

It is quite striking that most interventions do not affect both symptoms and workability. In some studies, only outcomes related to one of these were measured, but in the studies, both were reported (Volker et al. 2015; Arends et al. 2013; Klink et al. 2003; Vlasveld et al. 2012; Oostrom et al. 2009; Goorden et al. 2014; Noordik et al. 2013; Hees et al. 2013; Feltz-Cornelis et al. 2010; Lexis et al. 2011; Boer et al. 2004; Vuori et al. 2012), they would typically not follow each other. Thus, improving symptoms or diminishing the risk of CMD does not automatically improve workability (Vlasveld et al. 2012; Hees et al. 2013) and improvement in workability does not require an improvement in the patients’ CMD status greater than that of the control group (Arends et al. 2013; Klink et al. 2003). This finding may seem surprising, it is often inferred that when people are well, they will be able to work optimally. From this review we may conclude that for CMD, different mechanisms are determining symptoms and health than are determining workability. It seems that work-oriented CBT/PST interventions help individuals manage their work in such a way that they may be able to keep working despite still having CMD/ risk of CMD.

It is relevant to scrutinize the types of interventions offered in these studies. Commonly, they are targeting the individual, typically helping the individual to identify and manage stressors, largely ignoring any necessary action in the workplace. Even the interventions described as “workplace interventions” typically entails a meeting between the worker, the employer and a health care provider and targets barriers to RTW, i.e. individual adaptation (Vlasveld et al. 2012; Goorden et al. 2014; Dalgaard et al. 2014). Evidence is mounting that the psychosocial work environment is vital for the mental well-being of workers, yet many interventions fail to address this issue (Theorell et al. 2015; Aronsson et al. 2017).

There is clearly a need for evaluating theory-based preventive strategies with scientifically robust methods, such as the vital use of groups for comparison, to understand the effectiveness of such interventions. However, complex organizational interventions may also apply other methodological approaches to investigate their outcome, e.g. by a more detailed study of process mechanisms (Nielsen 2017). Only a few studies suggest that prevention among employees at risk is effective, but the interventions are diverse, so replication of these results is necessary. It may also be beneficial to combine these individually oriented interventions for employees at risk with more comprehensive organizational approaches (Kwak et al. 2019).

The review was done systematically in two rounds, in 2014 and again in 2017. Between the two searches, only 12 eligible studies were published. This alone is signifying the scarcity of evidence in the field but is also highlighting the fact that OHS is not an indexed term, making hand searches of reference lists necessary. Even though we used an experienced librarian and embarked on a systematic approach, searching the major databases, relevant studies may have been missed. A limitation of our review is that the first screening of abstracts in each round was done by one researcher only, although a calibration between the pair was undertaken and discussions ensued in cases of uncertainty. It is possible that the eligibility criteria were applied differently between researchers, possibly missing relevant articles. Further, conclusions from the grey literature were deliberately not included in our review, owing to the scientific weight of such evidence.

We purposefully only included studies where the OHS was involved in the intervention. This means that other important workplace interventions that were not labelled as such, or that were delivered by other parties, were not included in the review. The included studies also reflect the difference in OHS around the globe: nearly half of the eligible studies were from the Netherlands (Volker et al. 2015; Arends et al. 2013; Rebergen et al. 2009; Klink et al. 2003; Vlasveld et al. 2012,2013; Hees et al. 2013; Feltz-Cornelis et al. 2010; Boer et al. 2004), where the OHS is an important actor in worker health, supported by a legal framework. Only four included studies were from Asia (including a systematic review with a majority of articles from the Netherlands (Doki et al. 2015), and none were from the US, South America or Africa. This fact limits the generalizability of our findings outside the European and Asian setting. However, if similar services are offered in primary care in areas where OHS are not common, the results may be transferrable to these settings.

To conclude, there is some evidence that PST interventions or work-related CBT given at the OHS can improve workability among employees on sick leave due to CMDs but the evidence for these interventions concerning symptom reduction is uncertain.

## A summarized description of the included studies

### Studies reporting rehabilitation

In a RCT by Van der Klink et al. (2003), 4–5 sessions with the OHS physician were given early in the sickness absence to identify and solve stress issues. The control group also met the OHS doctor for treatment as usual (reflective listening, advice on stress and discussions centered on the work situation). After 12 months, the intervention group had less sickness absence in total and a quicker partial RTW, whereas no significant differences in symptoms could be found.

Rebergen et al. (2009) evaluated an intervention based on gradual activation and PST. The control intervention included minimal OHS physician contact but possible referral to a psychologist. After 12 months, time to RTW and production loss were equal between the groups, with less health care costs for the intervention group due to a frequent use of psychological treatment in the control group.

In a RCT by Arends et al. (2013), the PST intervention was given by OHS physicians after RTW to minimize new episodes of sickness absence. The control group got treatment as usual. After 12 months, the intervention group showed less recurrent sickness absences, although symptoms were equal in both groups. However, the intervention was not cost effective.

Vlasveld et al. (2012,2013) evaluated an intervention of 6−12 sessions of PST together with self-help manual and a meeting between the employee, employer, health care coordinator, and OHS physician. The control group got treatment as usual. After 12 months, the intervention resulted in shorter time to halving their depression symptoms, but there was no difference in time to RTW.

Goorden et al. (2014) performed a cost-effectiveness analysis on Vlasveld’s study above. After 12 months, the intervention group showed higher costs.

In a cluster RCT by Volker et al. (2015), an on-line CBT- and PST-based intervention related to work was supervised by the OHS physician. The control intervention was treatment as usual as recommended by guidelines. After 12 months, the intervention group had shorter time to first RTW, but no difference in full RTW or reduction of depression.

Doki et al. (2015) conducted a meta-analysis of PST or CBT interventions compared to treatment as usual. When pooling the data, the interventions led to a significant reduction of total sick leave.

In a RCT by de Vente et al. (2008), all participants were given stress management training based on CBT. Participants in one group then talked to a psychologist individually, and the participants in the other group met with the psychologist in a group session. The control intervention was care as usual as delivered by the OHS physician. After 4, 7 and 10 months, symptoms and sickness absence were equal between all groups.

In an observational study by Grossi and Santell (2009), an intervention with sessions regarding stress, cognitive strategies, Qigong and life-style. The control intervention was treatment as usual. After 6 months, less symptoms and sickness absence were reported by participants in the intervention group, but after 12 months, no differences were observed. After 5 years, sick leave was equal between the groups.

In a RCT, Dalgaard et al. (2014) investigated the effects of a CBT-based stress management program on sleep and cognitive failure. The intervention also included meetings between the employee, the employer and the psychologist. After 10 months, no difference between the groups was observed.

Hees et al. (2013) evaluated an extensive occupational therapist intervention including problem definition and problem- solving strategies with the employer. The control group was treated as usual. After 18 months, no differences in RTW were observed, although symptoms had diminished in the intervention group.

Van der Feltz-Cornelis et al. (2010) investigated whether the addition of a psychiatric specialist would improve the outcome. The control group was treated by the OHS physician as usual. After 3 months, the intervention group experienced a higher RTW, but after 6 months, there was no difference in symptoms or RTW.

In a study by Van Oostrom et al. (2009, 2010), a participatory intervention including a rehabilitation coordinator and the work manager. The control group received care as usual. After 12 months, there was no difference between the groups in RTW or symptoms.

In a study by Noordik et al. (2013), participants made a list of stressful events with the assistance of the OHS physician. The participants then exposed themselves to these situations in a graded manner. The control group got care as usual as recommended by guidelines. After 12 months, time to full RTW was shorter in the intervention group, although no difference was noted in symptoms.

Bender et al. (2016) examined the effect of an intervention with an educational program and RTW coordination. Participants in the control group were expected to seek care through the usual health care channels. In this study, the control group showed less sickness absence compared to the intervention group, possibly due to higher levels of depression in the intervention group.

In a cluster RCT by van Beurden et al. (2017), OHS physicians’ adherence to guidelines was investigated. The Dutch guidelines recommend a stepwise approach with problem-identification and contact with the employer, and the doctors were educated in a peer-trainer process. In van Beurden et al. (2015) [28], the participants in the intervention group had an increased belief in their RTW after 3 months. When examining the effect on sickness absence after 12 months, no difference between the intervention and cntrol groups was detected.

In a study by Kröger et al. (2015), participants were treated in a specialist clinic with CBT, either on its own (control intervention) or with the addition of a workplace intervention (intervention). After 12 months, participants in the intervention group had less sickness absence, however, no difference in the report of symptoms.

Valtonen et al. (2015) investigated an extensive multi-disciplinary intervention. The control group received treatment as usual. After 12 months, no differences were detected in participants’ sense of coherence.

### Studies directed at individuals at risk of CMD.

In a RCT by De Boer et al. (2004) an intervention aiming at decreasing early retirement among employees aged 50 years or older due to stress. The OHS physician individually met employees to identify and suggest strategies for stress management. After 2 years, the proportion of early retirements had decreased in the intervention group. No differences were noted in workability, sickness absence, burnout or quality of life.

Gärtner et al. (2013) and Ketelaar et al. (2013) evaluated an intervention where OHS physicians individually discussed symptoms of CMD, private and professional reasons for CMD and possible strategies to deal with it. The control group were treated as usual. After 6 months, the participants in the interventions group reported improved workability, but no difference in symptoms was detected. The intervention group also reported less use of health care resources.

In two studies by Noben et al. (2014, 2015), the economic consequences of the above study were investigated. In Noben et al. (2014), a cost-effectivene analysis from a societal perspective was reported. After 6 months, the intervention was more cost effective than care as usual. In Noben et al. (2015) an organizational perspective evaluates a cost-benefit analysis and concludes that the intervention is effective.

In a RCT, Kant (2008) also studied consultations with OHS physicians regarding symptom and private and professional stress. Further, the intervention included a discussion about the risk of future sick leave, as well as expectations towards the intervention. The control group was treated as usual and could consult the OHS physician as well. After 12 months, analysis excluding those who sought help on their own, showed that total sickness absence had diminished in the intervention group.

Kilfedder et al. (2010) and Karatzias et al. (2011) evaluated stress management in a RCT with (1) personal consultations and (2) consultations via telephone. The control group received bibliotherapy. After 4 months, the intervention groups experienced improvement in one measure but not in stress or CMD.

In a randomized study by Grime et al. (2004), employees in a county council underwent computerized CBT for 8 weeks. The comparison group received care as usual which could entail pharmacological treatment and/or psychological treatment. The intervention group (computerized CBT) experienced fewer depressive symptoms after 1 month. Three and 6 months after the intervention, no differences in depression or anxiety were seen between the two groups.

Lexis et al. (2011) examined CBT in combination with PST among employees in a bank. The intervention was planned as a seven-session program but could be prolonged with a maximum of five extra sessions, aimed at reducing sickness absence and symptoms of depression. Compared to the control group (which received usual OHS care including the possibility of consulting an OHS physician as needed), the intervention group had less sickness absence after 12 months as well as less symptoms of depression after 6 and 12 months (even though the study had a high number of missing data regarding depression). After 18 months, no difference between the intervention and control was found regarding sickness absence.

In a Japanese study from an IT-company by Yamamoto et al. (2016), workers were screened regarding insomnia with Athens Insomnia Scale. Those at risk of insomnia were randomized to an intervention or a waiting list. The intervention was a CBT sleep-school led by the OHS physician, given in a group (one occasion) and individually (three occasions). The primary outcome was change in emotional stress, measured at baseline and after 3 months with email. Secondary outcome was change in insomnia. No statistically significant difference between the groups was seen at 3 months.

In an observational study by Kuoppola et al. from Finland among employees in small- and medium-sized companies, an intervention based on mindfulness and meditation was evaluated (Kuoppala and Kekoni 2013). This intervention was given to all employees and comprised of five meetings and a follow-up. The development of symptoms of stress among employees with high levels of stress at baseline was compared with that of employees with less stress. After 6 months, the development of depression and burnout was better among those with high levels at baseline compared to those with low levels of stress. No difference was found between the groups regarding anxiety or burnout.

In a Swedish randomized study by Peterson et al. (2008), the effect of collegial therapy groups on health was on health care workers with symptoms of burnout. These groups discussed and reflected upon the reasons for burnout, formulated problem-solving and individual goals, and group members were supportive of each other. The intervention lasted for ten meetings and was led by personnel from the OHS. After 12 months, a positive effect on general health was seen in the intervention group compared to the control group, but no effects were seen on burnout, anxiety or depression.

Salmela-Aro et al. (2004) examined two types of interventions among employees who were in touch with the OHS regarding symptoms of burnout. One group was randomized to psychoanalytical therapy including free associations to create a safe environment for the participants to process feelings related to difficult situations such as workplace conflicts. In the second intervention group, the participants used psychodrama and sociodrama. Both interventions contained 16 full day sessions every other week. In a control group, participants were offered a consultation with a physician or a psychotherapist every other month. After 12 months, the intervention groups had fewer symptoms of burnout compared to the control group. Both the intervention groups also showed a decrease in personal work-related activities, which was considered positive in the risk of developing burnout.

### Preventive interventions

In a randomized study by Vuori and Ahola (2012) and Ahola et al. (2012), employees (with and without depressive symptoms) employed by the municipality were given a course to strengthen their own capabilities. The control group received written information concerning career advancement. After 7 months, the intervention group showed fewer depressive symptoms, increased mental resources and a decreased risk of sickness or retirement pension. However, when only analyzing the people with depressive symptoms, no statistically significant effect could be seen.

An observational study from the manufacturing industry by Kobayashi (2008), studied an intervention of employees participating in a program about participation and systematic work-environment action plans. The comparator was working units that did not get the intervention. After a year, women in the intervention group had less depression and increased vigor compared to the control group, but no such differences were seen among men.

Likewise, Kim et al. (2014) conducted an observational study in a metal industry, where all employees were involved in a stress-handling program. The program consisted of workshops for team leaders and for workers and administrators where action plans for improvements in the work environment were formulated. These action plans were then followed up after 2 months and a competition for the best work-environment improvement was launched. Stress was measured before and after the intervention, and work-related stress was found to decrease among workers, but not among administrators.

In a study by Vinberg (2015), administrators working in education and health care were subjected to several different individual and group-based interventions like physical activity, improvements in ventilation, health assessments, conflict handling, and teambuilding. A leadership course was also given to facilitate a new work schedule. Health and work environments were measured at baseline and after 1 and 2 years. Decreases in “tiredness at work” and “worry about ability to handle work tasks” were the only changes that were significant.
